# Influence of the teaching program on the learning in knowledge and practice of osteonecrosis of the jaws produced by antireasorptives in dental students of the Principality of Asturias (Spain)

**DOI:** 10.4317/jced.54129

**Published:** 2017-12-01

**Authors:** Matias-Ferrán Escobedo, Luis García-Consuegra, Silvia Gay, Lorena Álvarez, Sonsoles Olay, Giuliano Ascani, Luis Junquera

**Affiliations:** 1 School of Dentistry. University of Oviedo, Spain; 2 Dentist. Private practice, Asturias, Spain; 3 Department of Oral and Maxillofacial Surgery. Ospedale Civile dello Spirito Santo di Pescara, Italy

## Abstract

**Background:**

This study aims to evaluate the influence of changes in the teaching contents on medication-related osteonecrosis of the jaw may have on the knowledge and the capacity for practical case resolution about this pathology.

**Material and Methods:**

A cross-sectional descriptive study was conducted through a survey divided into four sections: degree of means of knowledge acquisition, habitual practice and ability to solve clinical cases. The total number of respondents (n = 225) was divided into two groups: Group A (Year 2015-2016) and Group B (Year 2016-2017). The students in Group B received more teaching content on the subject than group A.

**Results:**

A total of 175 survey responses were collected. The internet was the preferred tool for continuing education in both groups. The best known bisphosphonates (BPs) were Alendronate (Fosamax®: 56.9% Group A, 67.5% Group B) and Zoledronic Acid (Zometa®: 56.9% Group A, 51.8% Group B). A low percentage of students (37.9% Group A, 43.4% Group B) acknowledged the existence of other drugs that could also cause osteonecrosis of the jaws. Regarding the correct resolution of practical cases, the respondents of Group B reached a significantly higher score (5.67) than the score observed in Group A (4.04).

**Conclusions:**

Training on medication-related osteonecrosis among dental students is susceptible to improvement. Introducing minor changes in the teachings allows this goal to be successfully achieved.

** Key words:**Osteonecrosis of the jaw (ONJ), bisphosphonate-related osteonecrosis of the jaws (BRONJ), medication-related osteonecrosis of the jaw (MRONJ), dental education.

## Introduction

In September 2003, the first case series (36 patients) was published in the United States (1), which showed a relation between the use of aminobisphosphonates and the occurrence of bone exposure in the jaws. Since then, different terms have been coined (2): bisphosphonate-associated osteonecrosis of the jaws (BAONJ), bisphosphonate-related osteonecrosis of the jaws (BRONJ), bisp-hosphonate-induced osteonecrosis of the jaws (BIONJ), bisphosphonate-related osteonecrosis (BRON), or simply bisphosphonate osteonecrosis (BON). In the last thirteen years, a great number of works have been published in international literature about BRONJ in different countries around the worl. In Spain, the first case series was published in 2005 by Bagán *et al.* (3) on 10 patients in the Valencian Community. All were advanced stage cancer patients who had received different chemotherapeutic agents along with zoledronic acid and / or pamidronate. Subsequently, numerous references of BRONJ appeared in the literature on patients with osteoporosis who had been taking aminobisphosphonate orally, although the risk of BRONJ is lower than in patients who are intravenously treated with zoledronic acid (4,5). In 2007, the HORIZONT PTF (6) clinical trial recognized the efficacy of annual intravenous doses of zoledronic acid (5 mgr) for the treatment of patients with osteoporosis. Although BRONJ is not reported as having adverse effects on the treatment in this study, few papers have recently been published regarding this complication (7,8).

In 2014, the American Association of Oral and Maxillofacial Surgeons (AAOMS) (9) confirmed the relation of other drugs (Denosumad, Sunitinib, Sorafenib, Bevacizumab, Sirolimus, and others) dissimilar to BPs with the occurrence of chemical osteonecrosis of the jaws. Therefore, the concept of BRONJ has now been changed by the concept of medication-related osteonecrosis of the jaw (MRONJ).

The frequency of MRONJ is anecdotal for different specialists who prescribe these drugs (urologists, gynecologists, traumatologists, rheumatologists, primary care physicians, oncologists and those responsible for bone and mineral metabolism units). Even some general dentists question the true existence of MRONJs, even though they are one of the main components in their prevention.

In 2015, Alhussain *et al.* (10) published a paper whose findings concluded that general dentists and specialists in Ontario have an appropriate knowledge of BRONJ, but most are not comfortable performing oral surgery in patients taking BPs. Those who are comfortable rank higher in knowledge scores, suggesting greater educational efforts should be made to promote the knowledge of dentists regarding this complication.

This study aimed to evaluate whether changes in the teaching contents on medication-related osteonecrosis of the jaw led to changes in the knowledge of the surveyed students, quantifying whether the problem-solving capacity to carry out dental treatment on patients with MRONJ is significantly different according to the educational modifications introduced. Ethical approval was obtained from the University of Oviedo to conduct this study.

## Material and Methods

Study design

A cross-sectional descriptive study was carried out through a survey based on the proposal made by Alhussain *et al.* (10). This survey was modified by dividing it into 4 parts: questions on MRONJ knowledge, questions on MRONJ knowledge acquisition, questions on standard practice, on complication and a final section on case studies on oral surgery, implant therapy, periodontal treatment (scaling and root planning) and endodontic treatment. Each of the items on these sections had four possible answers. The student who was capable of correctly answering the clinical questions obtained a score of 12. The validation of accurate solutions was made by consensus between five experts, (three oral surgery specialists, one oral medicine specialist, and one bone and mineral metabolism specialist), taking as a clinical guide the proposal established by the AAOMS in 2009 (11). The students were divided into two groups. The first group (Group A) was made up of students from the 1st to the 5th dentistry courses that did the survey in November, 2015. The second group (Group B) was made up of those students (from the 1st to 5th) who carried out the same survey in November 2016. In turn, both groups were divided into two preclinical groups (1st and 2nd year) and students of clinical course (3rd, 4th and 5th year). The difference between Group A and Group B object of this study was the change in teaching contents. The students in Group B received more training on the MRONJ than those in Group A. In particular, the contents of this area increased by four hours. One hour during the 2nd year on the subject of Pharmacology, two hours during the 4th year on the subject of Oral Surgery II and, finally, one hour in the 5th year on the subject of Integrated Dentistry.

All data were made anonymous and transferred to a database, using the statistical software SPSS for Windows version 15.0. A descriptive analysis of variables was performed. All categorical variables are presented as percentages and continuous variables as mean and standard deviation. Student t test was used for comparison in case of quantitative variables, and chi-square or Fisher exact test in case of categorical variables. To compare more than two groups of quantitative values, ANOVA test and the Bonferroni’s post hoc procedure were used. Probability of less than 0.05 (*p*< 0.05) was accepted as significant.

## Results

A total of 175 surveys were collected (77.7%) from the total sample (n = 225 students: 125 Group A and 100 Group B). The age range of the students in the sample was 18 to 28 years old and 66.1% of the respondents were women.

1. Knowledge acquisition.

The most widely used resource to be up-to-date on the subject was the Internet (85.2% Group A, 84.3% Group B), and secondly, specific courses on the subject (23.9% Group A, 36.1% Group B).

2. Degree of knowledge.

Less than 20% of students (19.6% of Group A, 19.3% of Group B) stated that they had no knowledge of BPs. Seventy-five percent of respondents reported that they received the first information on these drugs and their complications in the graduate program at University. The most well-known bisphosphonates were Alendronate (Fosamax®: 56.9%, Group A, 67.5% Group B) and Zoledronic Acid (Zometa®: 56.9% Group A, 51.8% Group B) ( Table 1).

**Table 1:**
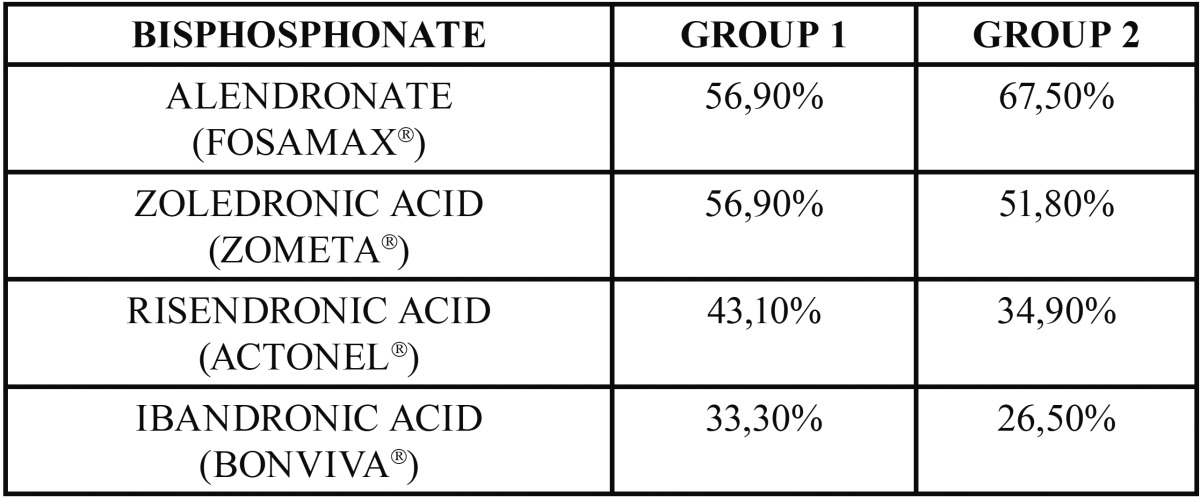
Knowledge percentage of about different bisphosphonates by the respondents.

Regarding BPs indications, 84.8% of the students in Group A and 81.9% of those in Group B indicated that they were used for the treatment of osteoporosis. A lower percentage, (38% Group A, 44.7% Group B), they were also used for the treatment of patients with bone metastases ( Table 2). 91.4% (Group A) and 94% (Group B) referred oral administration. The use of intravenous administration (IV) was recognized in 82% of respondents. In Group A, 80% of the students did not know the treatment by stages of osteonecrosis, 73% of the respondents of this group admitted to not knowing some type of treatment protocol. On the other hand, in Group B, the percentage of students who were unaware of treatment by stages was lower (50.6%), although 61.4% said they did not know any treatment protocols. A relatively low percentage of students (37.9% Group A, 43.4% Group B) recognized that other medicines other than BPs may cause osteonecrosis of the jaws. Denosumab was known for 20.5% and 39.8% of Group A and Group B, respectively. On the other hand, 74.2% (Group A) and 60.2% (Group B) were unaware of the relation between Sunitinib and osteonecrosis of the jaws.

**Table 2:**
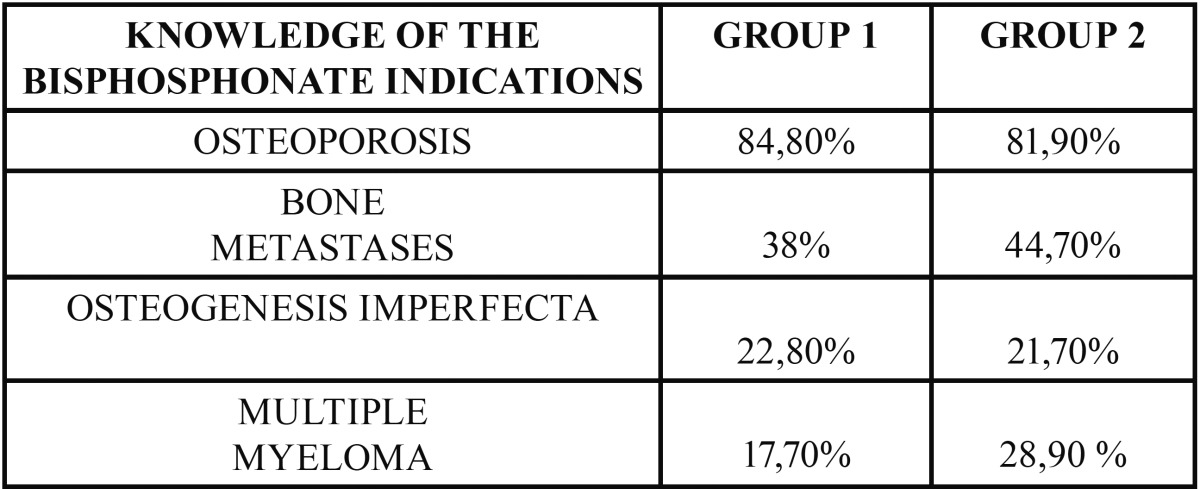
Knowledge of the bisphosphonate indications, reported by the two study groups.

3. Common practice.

As expected, given the provenance of the study sample, more than 80% of the respondents had not treated any patient receiving BPs and more than 90% had not had a chance to see the treatment of patients with established osteonecrosis.

Case resolution. ( Table 3)

**Table 3:**
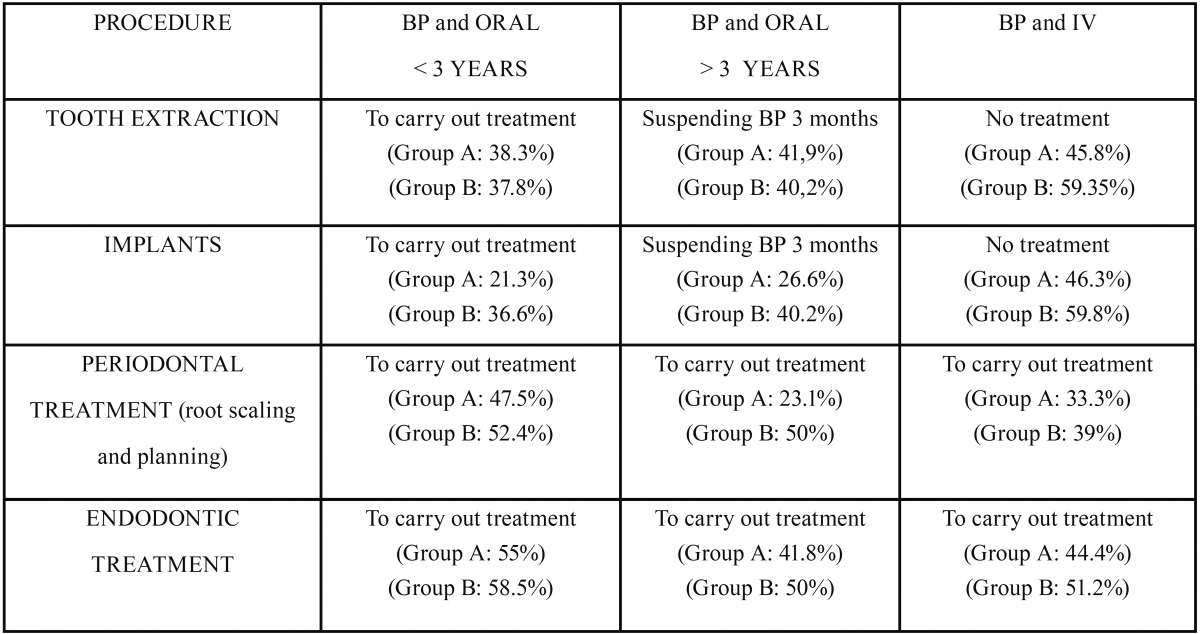
Guideline of performance for dental procedures in patients to BP treatment and percentage of right answer in the respondents. O: oral Administration, IV: intravenous administration.

Tooth extraction

Right Answer: 45.8% (Group A) and 59.3% (Group B) of student would not carry out the treatment when the BP was administered by IV, while 38.3% (Group A) and 37.8% (Group B) would carry out the treatment when BP was orally administrated for less than three years. About 41% would stop BP 3 months before performing dental extraction when BP was taken orally for more than 3 years.

Implants

Right Answer: 46.3% (Group A) and 59.8% (Group B) of students would not carry out dental implants when the BP was administered by IV and only 21.3% (Group A) and 36,6% (Group B) would carry out treatment when BP was taken orally for less than three years. Before placing an implant, 26.6% of Group A respondents would stop oral treatment when BP was taken for more than three years. This same attitude would be applied by 40.2% of the students in Group B.

Periodontal treatment (root scaling and planning).

Right Answer: 33.3% of the students in Group A and 39% of the students in Group B would perform a periodontal treatment (root scaling and planning) when BP was administered by IV. 47.5% and 52.4% respectively, would carry out this same treatment when BP were taken orally for less than three years and only 23.1% and 50% (Groups A and B respectively) would do so when BPs were taken orally for more than three years.

Endodontic treatment.

Right Answer: 44.4% of Group A and 51.2% of Group B respondents would carry out an endodontic treatment, when BP was taken by IV. 55% and 58.5% (Groups A and B, respectively) would carry out endodontic treatment when BPs were given orally for less than three years. Only 41.8% (Group A) and 50% (Group B) would carry out endodontic treatment when BPs was taken orally for more than three years.

Considering that the maximum score to be obtained is 12 points and the minimum score is 0, the average score obtained by the 92 students in Group A was 4.04 with a standard deviation (SD) of 3.46. In Group B, 83 students obtained an average score of 5.67 with a SD of 4.40. Significantly, students in Group B had more right answers in the correct treatment related to the tooth extraction of the patients who received intravenously bisphosphonate (*p*= 0.03), implant surgery in patients who had been taking oral bisphosphonates (*p*= 0.02) and the appropriate periodontal treatment of patients who had been taking oral bisphosphonates for more than three years (*p*= 0.001). The difference in the final score reached in the resolution of practical cases (4.04 in Group A versus 5.67 in Group B) was statistically significant (*p* = 0.001).

Subdividing the study groups into two blocks (preclinical and clinical) according to the course of study at the time of the survey allowed us to observe that the average score of first and second year students in Group AP (preclinical) was 1.39. On the other hand, the average score of the third, fourth and fifth year students reached a value of 5.02. ( Table 4).

**Table 4:**
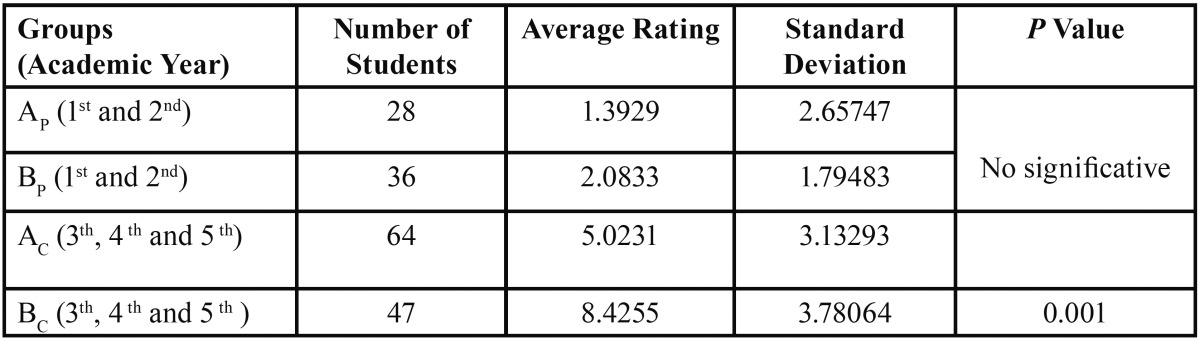
Inferential analysis between Groups A and B according to academic course. p: preclinical. c: clinical.

For the students in Group BP (preclinical) in the first and second year the average score reached was 2.08, significantly lower than the score obtained by the students in the last years of training (8,12) ( Table 4). We did not observe differences between scores achieved by students of Group A compared to those of Group B in the first years of study, but there were differences among the students of last years. Significantly those in Group B achieved a higher score.

Among respondents in two study groups (A and B) there were no significant differences between variables, age, sex, Internet preference for knowledge acquisition, or importance of scientific journals as a source of knowledge. However, denosumab (Prolia®) was significantly better known in Group B students in regard to those of Group A (*p* = 0.01).

## Discussion

To the best of our knowledge, López-Jorner *et al.* (12) published the first work in 2010 with similar characteristics to our study. They compared knowledge about BRONJs among students and dentists in Murcia. As in our work, they observed that the Internet was the main source of knowledge for both groups, and that alendronate was the best known bisphosphonate. In their results, the knowledge of dentists about BRONJs was higher than the student´s knowledge. In their opinion, it would be necessary to increase training on BRONJ in pre and postgraduate training.

In 2015, de Lima *et al.* (13) published the second work of similar characteristics carried out between dentistry students and dentists in Pernambuco (Brasil). Alarmingly, they observed that 84.6% of dentists and 86% of dental students did not know the commercial names of bisphosphonates with the importance that this lack of knowledge has to make a correct medical history. 65.4% of dentists (most of them generalists) and 54% of students did not know the indications of bisphosphonates. Dentists who had completed their studies more recently (less than five years of the survey) had better knowledge about BRONJ than dentists with more work experience.

In general, all of the literature reviewed agrees on the need to increase MRONJ training among dentists and this was the main objective of the present study. For this purpose, the survey model documented in Canada by Alhussain *et al.* (10) was modified, gathering information on the capacity for practical case resolution including different areas of dental treatment. This information allowed us to obtain quantitative assessments on the influence of changing teaching contents on bisphosphonates, antiresorptive and osteonecrosis in dental students. In our view, the first step in improving MRONJ training should begin during undergraduate studies. Appropriate training in the management of these patients at the end of dentistry studies should be one of the main objectives to achieve an improvement in the reduction of MRONJ cases. The introduced teaching variable is easily reproducible in any educational center. This variable only consisted in the increase of teaching hours on MRONJ.

The present study emphasizes that we must improve our teaching efforts, since it shows that more than half of students do not know any action protocol to follow for patients receiving these medicines. Regarding knowledge of a guide to the treatment of MRONJ, the American Society of Oral and Maxillofacial Surgery (AAOMS) (9,11) was the best known, but only by 15.7% of respondents. Another data obtained from the study to be taken into account was that a low percentage of respondents (37.9% Group A, 43.4% Group B) recognized that other drugs (denosumab) can cause osteonecrosis of the jaws. However, denosumab was significantly better known among Group B students compared to Group A.

In practical case resolution, significant differences were observed in favor of Group B in the activities related to surgery, (tooth extraction, implants) and to a lesser extent in some cases of periodontal treatment. This makes us think about the need to implement training on the management of these patients in periodontal and endodontic treatments.

In conclusion, our study highlights the need to improve teacher training of dental students on MRONJ in our context. This study also demonstrates that small teacher modifications are accompanied by significant improvements in the clinical management of this complication.
